# The disease burden of suicide in Ecuador, a 15 years’ geodemographic cross-sectional study (2001–2015)

**DOI:** 10.1186/s12888-017-1502-0

**Published:** 2017-10-10

**Authors:** Esteban Ortiz-Prado, Katherine Simbaña, Lenin Gómez, Aquiles R. Henriquez-Trujillo, Fernando Cornejo-Leon, Eduardo Vasconez, Diana Castillo, Ginés Viscor

**Affiliations:** 1 OneHealth Research Group, Faculty of Medicine, Universidad De Las Americas, Quito, Ecuador; 2 Department of Cell Biology, Physiology and Immunology, Universitat de Barcelona, Barcelona, Spain; 3 Faculty of Medicine, Universidad Central del Ecuador, Quito, Ecuador; 4 Faculty of Medicine, Universidad Tecnologica Equinoccial, Quito, Ecuador; 5 One Health Research Group, Universidad de las Américas, Quito, Ecuador Calle de los Colimes y Avenida De los Granados, 170137 Quito, Ecuador

**Keywords:** Suicide, Self-inflicted injury, Pesticides, Hanging, Firearms, Ecuador, DALY

## Abstract

**Background:**

Suicide affects people from different backgrounds, ethnical groups, socio-economic status and geographical locations. In Latin America, suicide reports arescarce, specially in Andean countries. In Ecuador, very few reports have partially described this phenomenon, nonetheless, estimation of the burden of disease (BoD) hasnever been reported in the country.

**Methods:**

A country-wide comparison was performed using the Ministry of Public Health’s national databases of overall mortality, Hospital Discharges Database, and the Population Census of the National Institute of Census and Statistics (INEC). The study variables analyzed were age, geographical distribution to provincial level, sex, means of suicide, educational attainment, marital status and mortality. Linear Regression and relative Risk analysis were used to predict outcome and the likelihood that suicide occur among study variables.

**Results:**

In the last 15 years, 13,024 suicides were officially reported. Men were 3 times more likely than women to die by suicide. The overall age-adjusted suicide ratio in Ecuador corresponds to 7.1 per 100,000 per year. The sex-specific rates were 5.3 in women and 13.2 in men. The primary mean of suicide was hanging X70 (51.1%), followed by self-poisoning X68-X69 (35.2%) and firearms X72-X74 (7.6%). Provinces located at higher altitude reported higher rates than those located at sea level (9 per 100,000 vs 4.5 per 100.000). The total economic loss due to suicide was estimated to be $852.6 million during the 15 years’ analysis.

**Conclusions:**

This is the first geodemographic study exploring the complete burden of suicide in Ecuador and one of the very few in Latin-America. In the last 15 years of available data, Ecuador ranks above the regional average with an adjusted suicide rate of 7.1 per 100,000 inhabitants. An important finding is that Suicide affects rather younger populations, adding more than 10,000 years of premature years of life lost (YYL) between 2001 and 2015, becoming the first and fourth leading cause of death among adolescent women and men respectively.

Suicide affects people from different backgrounds, socioeconomic status and educational attainment. The mean of suicide changed over time showing that gun and pesticides related deaths decreased significantly since 2001, while hanging and suffocation increased in more than 50%.

## Background

Suicide affects people from different backgrounds, ethnical groups, socio-economic status and geographical locations [1–3]. Suicide can be defined as any deliberated act by which an individual’s death results directly or indirectly from a self-inflicted injury or poisoning [4, 5]. Suicide is a global health problem, ranking between 4th to 20th cause of mortality for any given region in the world [6, 7]. According to the World Health Organization (WHO),a person commits suicide every 40 s [1]. In 2012, suicide accounted for 1.4% of all deaths worldwide, the majority occurring in low- and middle-income countries [8, 9].

Suicide rates vary across countries, ranging from less than 1 per 100,000 deaths in Saudi Arabia and Belize to more than 40 per 100,000 deaths per year in Guyana or Lithuania [1, 10]. The Global Burden of Disease Group (GBD) reported that in 2013, more than 840,000 people died from self-harm worldwide [7]. According to this report, suicide was the 14th leading cause of years of life lost in the world [7].

Suicide is a multifactorial problem influenced by genetic, psychological, cultural and environmental factors [11].

Several risk factors for suicide have been clearly documented, e.g. male sex, younger age, having a psychiatric disorder such as depression and schizophrenia, hopelessness, marital status, white race, low income, owning firearms, incarceration, job loss and drug abuse [12–18]. Recently, a positive association between altitude gradient and suicide has been described [19].

It is estimated that about 30% of suicides in the world are committed by poisoning with pesticides, hangings or firearms, and the means of suicide depend greatly on the availability and access to the lethal object used by the person [20, 21].

In Latin America, the highest mortality rate comes from Uruguay; a small country with a little more than three million people reported more than 15 per 100,000 deaths per year from suicide in 2012 [6, 22]. Other countries like Guatemala (2.5/100.000), Venezuela (3/100.000) or Peru (5.8/100.000), reported more lower rates, averaging 3.7 per 100,000 deaths in 2012 [6].

In South America suicide rates change according to cultural, behavioral and socioeconomic factors, however, data availability and under-ascertainment might also contribute to these differences. Adding up all the regional reports, it was estimated that more than 45,800 people commit suicide every year in this region [23].

Data about suicide is still scarce in Latin America, especially in smaller countries like Ecuador. In the last 20 years just very few reports have partially described this phenomenon in the country [24–26].

The Observatory for Citizen Security in Quito carried out a study on the subject and determined that in 2014 in Ecuador there were 9 suicides per 100,000 inhabitants with similar results in 2015 [27]. The most recent scientific reports on Ecuadorian suicide epidemiology comes from 2011, with two descriptive analyses that offer an interesting demographic analysis of the problem, however, none of those reports included a detailed geodemographic and economic analysis of suicide, years of life lost, disability-adjusted life years (DALY) or risk analysis [24, 26].

In this sense, this document responds to the lack of data about the economic and public health impact of suicide in Ecuador, adding new insights about a problem that has been poorly described in Latin American countries will improve the capacity of policy makers to correctly approach this health problem.

## Methods

### Study design and population

The present study is an observational cross-sectional analysis of suicide data and the burden of disease in Ecuador in the last 15 years of available data, from 2001 to 2015.

Ecuador is situated in South America and according to national census data, the total population surpassed 16.4 million people in 2015 [28].

A country-wide comparison of the data from the 24 provinces was performed using the complete dataset from the Ministry of Public Health’s national databases on overall mortality and hospital discharge data from 2001 to 2015, as well as 2010 population census data from the National Institute of Census and Statistics (INEC). The study variables analyzed were age, geographical distribution at the provincial level, sex, means of suicide, educational attainment, marital status, mortality and high altitude exposure.

### Data sources and description

All cases of suicide were classified according to the International Classification of Diseases 10th Revision (ICD-10). ICD-10 codes reflecting intentional self-harm (X60-X84) were identified through autopsy reports provided by INEC. Data on demographic variables such as sex, province elevation, educational attainment and marital status for all cases of suicide were included.

The burden of disease attributable to deaths by suicide in Ecuador was measured in disability-adjusted life years, or DALYs. [29]. DALYs are the sum of years lived with disability (YLDs) and years of life lost due to premature mortality (YLLs). In the case of suicide, only YLLs were considered for the calculations. YLLs were computed as the number of deaths multiplied by life expectancy at the age of death for specific age and sex strata considering a life expectancy at birth of 80 years for men and 82.5 years for women. A standard time discounting of 3% without age weighting was used. All calculations were made in the “DALY” package for R [30].

### Data analysis

The demographic variables, suicide method (X60-X84), place and date of suicide as well as the DALY data was analyzed using descriptive statistics. Data are presented as mortality rates, absolute values and proportions. The mortality rate was adjusted by age, sex and geographic location. Linear Regression and relative Risk analysis were used to predict outcome and the likelihood that suicide occur among variables. The calculations were completed using the IBM SPSS statistics version 24.0. References, citation and retrieval were managed by Zotero Open Source Software version 4.0.11. Spatial analysis was performed using QGIS 2.8 and the graphics were designed using the Piktochart infographic online app.

### Ethical considerations

According to the international guidelines of good clinical practices (GCP) and the Helsinki Declaration, anonymous databases can be used when no harm or confidentiality can be guaranteed [31]. According to the national guidelines we have complied and met the criteria described above and the study approval was exempted by the BioEthics Committee for Human Research at Universidad de las Americas (CEISH-UDLA) [32]. The de-identified non- biological data is freely available to the public at the following link www.inec.gob.ec and is owned by the Government of Ecuador, through the National Institute of Statistics and Census of Ecuador (INEC).

## Results

In Ecuador, over a period of 15 years from 2001 to 2015, a total of 13,024 deaths were attributed to self-inflected injuries. Over this time period, suicide rate fluctuated between 4.3 to 7.1 per 100.000. The overall national suicide mortality rate in private and public healthcare systems averaged 7.5 per 100,000 deaths. Men were 3 times more likely than women to die by suicide. The highest rate of suicide was recorded in Carchi province (12.7 per 100,000) and the highlands provinces had higher rates than those located at sea level (9 vs 4.5 per 100.000). For the first time in history, the rate of suicide in 2015 (7.9 per 100,000) surpassed the homicide rate (6.3 per 100,000).

### Age distribution

**Fig. 1:**
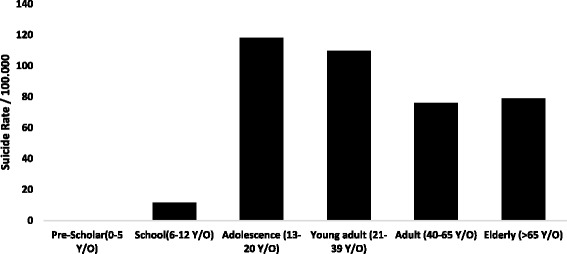
Suicide rate per 100.000 by age in Ecuador from 2001 to 2015 Source INEC

Suicide in Ecuador affects all age groups, but adolescents and young adults are the most likely group to commit suicide (Fig. 1). During the 15 years of analysis, 262 children younger than 12 years old committed suicide; Azuay province had the highest cumulative percentage reported for this group (4%).

Among adolescents, suicide ranked first among all the causes of death in women aged 13 to 20 years old and 4th in men in the same age group, accounting for more than 17% and 10% of mortality, respectively (Table 1.).

**Table 1:** Main causes of mortality among men and women adolescents between 2004 and 2015

Adolescent Men 13–20 years			Adolescent Women 13–20 years		
Cause of death	N	%	Cause of death	N	%
096 Road traffic injuries	3376	22%	101 Intentionally Injured Injuries	1217	17%
102 Aggression	2564	17%	096 Road traffic injuries	788	11%
103 All other external causes	1574	10%	103 All other external causes	541	7%
101 Intentionally Injured Injuries	1501	10%	094 Other findings not elsewhere classified	511	7%
098 Accidental drowning and submersion	776	5%	102 Aggression	352	5%
094 Other findings not elsewhere classified	702	5%	061 Rest of diseases of the nervous system	290	4%
045 Leukemia	465	3%	045 Leukemia	288	4%
061 Rest of diseases of the nervous system	407	3%	074 pneumonias	271	4%
074 pneumonias	397	3%	089 Other direct obstetric deaths	252	3%
068 Other heart diseases	322	2%	068 Other heart diseases	215	3%
Other causes	3197	21%	Other causes	2872	36%
Total	15,224	100%	Total	7365	100%

The proportion of suicide by age groups in relationship with all other causes of death from 2001 to 2015 was 2.9% in children (6–12 years), 12.0% in adolescents (13–20 years), 5.8% among young adults (21–39 years) 1.5% in adults (40–65 years) and 0.2% among the elderly (>65 years old).

### Sex differences

**Fig. 2:**
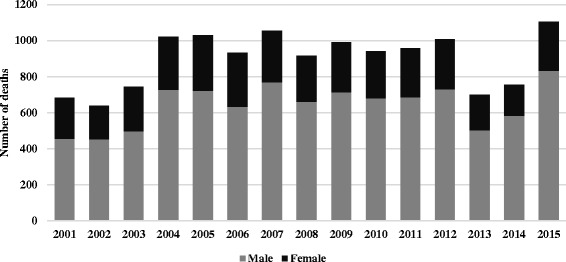
Sex Distribution of suicide in Ecuador from 2001 to 2015 Source INEC

In Ecuador, men account for a majority of suicides, 71.5%. The overall sex-specific rates in Ecuador is 5.3 for women and 13.2 for men (Fig. 2) and the men to women ratio is 3:1. The proportion of suicide in comparison with all other causes of death is 1.9 in men and 1.0 in women.

### Means of self-inflicted injury

**Fig. 3:**
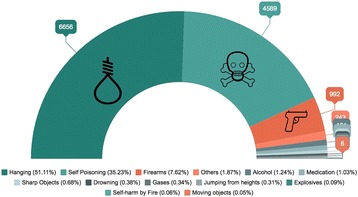
Means of Self-inflected injury from 2001 to 2015 Source INEC

The means used by persons who committed suicide in Ecuador differs according to sex and location of the victim. The principal means of self-inflected injury reported in the last 15 years is the X70: Intentional self-harm by hanging, strangulation and suffocation (ICD-10 X70), followed by Intentional self-poisoning by exposure to pesticides (ICD 10 X68) and then firearms (ICD 10 X72–74). (Fig. 3).

**Fig. 4:**
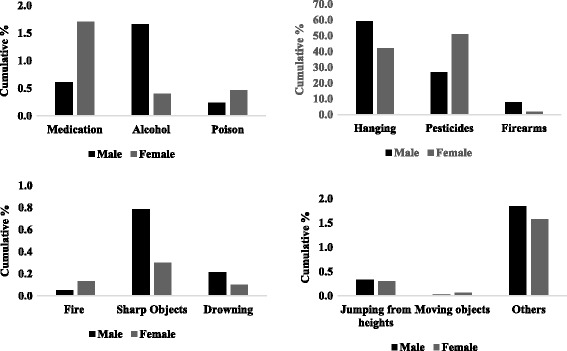
Means of Self-inflected injury from 2001 to 2015 Source INEC

Women are more likely to use less violent means, such as poisoning and drug abuse, while men are more likely to use hanging, firearms or intentional self-harm with sharp objects (Fig. 4).

**Fig. 5:**
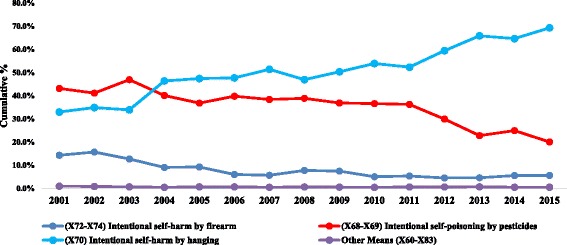
Cumulative percentage of the mean of suicide in Ecuador in the last 15 years 2001–2015

Self-inflicted deaths by firearms accounted for almost 15% of the of means of suicide s in 2001 and 5% in 2015. Hanging increased from 33% in 2001 to 69.3% in 2015 and pesticide self-poisoning decreased from 43% to 20% in the same time period (Fig. 5).

### Geographical differences

**Fig. 6:**
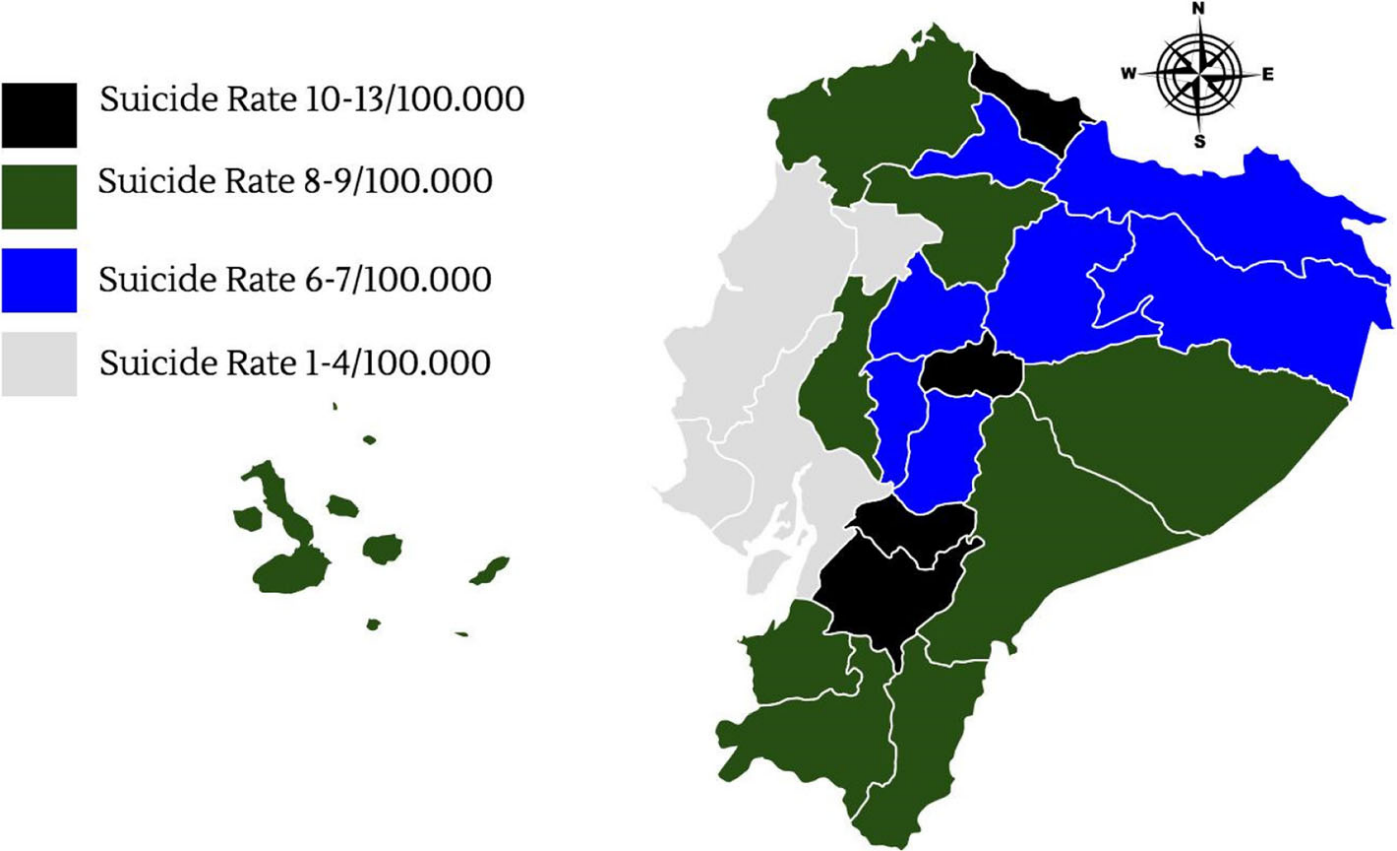
Geographic distribution of suicide rate in Ecuador from 2001 to 2015 Source INEC

The provinces with the highest suicide rate per 100.000 in Ecuador are Carchi (12.7/100,000), Napo (11.6/100,000), Cañar (11.3/100,000) and Azuay (11.0/100,000) (Fig. 6).

The lowest recorded suicide rate was reported in the coastal province of Santa Elena with 1.4 per 100.000. The highlands region of the country had the highest suicide rate (9 per 100,000), followed by the Amazonian region (8.1 per 100,000), the insular region of Galapagos (5.6 per 100,000) and finally the coastal region (4.5 per 100,000).

When the average elevation was analyzed, higher altitude locations (>2500 m) had a rate of 9.4 per 100,000 while lower altitude locations (<2500 m) had a rate of 6.3 per 100,000) (Table 2). We performed a logistic regression and a relative risk analysis for elevation (in meters) and suicide. The results show that living at high altitude has a very low chance to predict the occurrence of suicide (R^2^ = 0.0232, *p* = 0.477), however the relative risk (RR) analysis showed a slightly high increment in the risk of committing suicide when living above 2500 m (RR = 1.545, 95% CI: 1.4890 to 1.6055, *p* < 0.0001).

**Table 2:** Suicide Rate by province, region and elevation from 2001 to 2015 * deaths per 100,000

	Suicide rate	Region	Average Elevation/m	Altitude Classification
Azuay	11.0	Highlands *9/100,000	2560	High altitude > 2500 m *9.4/100,000
Bolívar	8.9		2668	
Cañar	11.3		2518	
Carchi	12.7		2980	
Cotopaxi	8.5		2800	
Chimborazo	8.3		2754	
Pichincha	5.9		2800	
Tungurahua	8.6		2577	
Imbabura	8.6		2225	Low Altitude < 2500 m *6.3/100,000
Loja	5.8		2060	
El Oro	5.4	Coast *4.5/100,000	6	
Esmeraldas	5.6		15	
Guayas	4.1		4	
Los Ríos	6.2		8	
Manabí	4.7		6	
Santo Domingo de los Tsáchilas	4.2		635	
Santa Elena	1.4		45	
Morona Santiago	6.8	Amazonia *8.1/100,000	940	
Napo	11.6		598	
Pastaza	6.1		790	
Sucumbíos	9.6		297	
Orellana	9.7		300	
Zamora Chinchipe	5.1		920	
Galápagos	5.6	Galapagos *5.6/100,000	6	

### DALYs and YLLs analysis

During the period 2001–2015 in Ecuador there were 13,024 deaths registered as self-inflected injuries causing death. These deaths are equivalent to 135,731 years of life lost due to premature mortality. 93% of YLLs are attributable to deaths in the economically active population. 73.6% of the burden generated by premature mortality occurred in men (Table 3).

**Table 3:** Years Life Lost (YLL) due to suicide in Ecuador, years 2001–2015*

Year	YLL Males	YLL Females	Total YLL	per 10,000 pop.
2001	4306.62	1927.21	6233.82	4.86
2002	4332.90	1853.19	6186.10	4.72
2003	4975.93	2249.54	7225.47	5.42
2004	6069.33	2379.26	8448.59	6.23
2005	7430.72	2669.09	10,099.81	7.36
2006	6564.18	3057.33	9621.51	6.89
2007	7939.52	2826.75	10,766.27	7.57
2008	7248.46	2676.84	9925.30	6.86
2009	7834.41	2542.20	10,376.62	7.04
2010	7173.49	2627.56	9801.06	6.53
2011	7108.09	2707.92	9816.01	6.43
2012	7657.87	2734.28	10,392.15	6.70
2013	5207.05	1903.13	7110.18	4.51
2014	6322.19	1787.59	8109.78	5.06
2015	8784.75	2834.24	11,618.99	7.14
Total	98,955.53	36,776.12	135,731.65	6.22 (±1.03)**

*Years Life Lost computed as the number of deaths times the life expectancy at the age of death for specific age and sex strata, considering a life expectancy at birth of 80 years for males and 82.5 years for females. A standard time discounting of 3% without age weighting was used

**Average YLL rate per 10,000 population (±SD)

### Sociodemographic characteristics

In Ecuador, the majority of people committing suicide are single, followed by married and common law couples. Educational attainment was found to be another important factor related with suicide in Ecuador. In the last 15 years, people who did not finish primary or high school had the highest suicide rates among Ecuadorians. Urban communities have almost triple the suicide rates of rural communities. The overall suicide rate by race shows that mestizos or mixed race has the highest rate (9/100.000), followed by indigenous (7/100.000) and all the other ethnic groups (2/100.000). Suicide rates tend to be higher during January, March and October as described in Table 4.

**Table 4:** Sociodemographic and monthly variation of suicide in Ecuador from 2001 to 2015

Civil Status	Cumulative %	Suicide Count	Month	Suicide Count	Cumulative %
Common-law	17.50%	2283	January	1292	9.9%
Single	48.00%	6248			
Married	23.20%	3025	February	970	7.4%
Divorced	1.90%	245			
Separated	1.90%	248	March	1087	8.3%
Widower	2.20%	291			
Unknown	5.30%	684	April	996	7.6%
Total	100%	13,024			
			May	1079	8.3%
Educational Attainment	Cumulative %	Suicide Count	June	1008	7.7%
illiteracy	5.70%	737			
Preschool	1.00%	130	July	1043	8.0%
School	48.50%	6323			
High School	31.40%	4087	August	1024	7.9%
Undergraduate	4.70%	611			
Graduate	0.10%	8	September	1066	8.2%
Unknown	8.70%	1128			
Total	100.00%	13,024	October	1105	8.5%
			November	1030	7.9%
Rural/Urban	Cumulative %	Suicide Count			
Urban	69.90%	9098	December	1016	7.8%
Rural	25.10%	3269			
Unknown	5%	657	Unknown	308	2.4%
Total	100%	13,024	Total	13,024	100%

## Discussion

The results from this investigation demonstrate that suicide is one of the most important health issues in Ecuador. Adequate public policy needs to be pursued in order to generate effective public health prevention programs. The results in Ecuador accompany and support the WHO global epidemics [10, 33]. Nearly 13,500 deaths were attributed and officially reported as suicide in the last 15 years in Ecuador, averaging almost 890 deaths per year, with an average suicide rate of 7.1 per 100,000. In Ecuador, men are three times as likely to commit suicide than women; this relationship of 3:1 seems to be within the range described worldwide [10, 22, 34].

Suicide rates vary from region to region, affecting provinces located in the Andean region of Ecuador more than those located at lower altitudes. It is not well understood why this relationship is present in Ecuador, however, other reports have also suggested the positive relationship between altitude and suicide rates [19, 35].

The differences between highland and lowland communities have been well documented and include cultural, physical or socioeconomic variances [27, 36]. Although there is not much research on this subject it is speculated that altitude can increase psychiatric disorders, mood changes, risk of depression and risk to develop stroke [37–40]. Greater access to firearms, isolation or reduced income have also been implicated as possible causes [35]. In Ecuador, high altitude provinces have more presence of indigenous groups than those provinces located at the coast. According to the latest National Census, this vulnerable population has less access to proper housing, education and health care [41]. The World Report on Violence and Health in 2002 found that suicide rates have increased among indigenous people [42]. Among the proposed risk factors, cultural differences, discrimination, poor adaptation to urban lifestyles, poverty and limited health access were identified [42, 43]. In this sense, Carchi and Azuay, two of the provinces with the highest suicide rates experienced a massive emigration from their young adults to the US and Europe in the year 2000 [44]. Immigration patterns are variables that increase the risk of suicide due to the apparition of broken families and children that were raised alone, make this group particularly vulnerable to depression, drug abuse and probably higher risk of suicide [45–47]. On the other hand our results might indicate that living at high altitude could be considered a non-traditional risk factor, although the effect of hypoxia on suicidal thoughts or behavior has not been demonstrated yet, a slightly higher relative risk of committing suicide was demonstrated among high altitude dwellers when compared to those located at lower altitudes [19, 35].

Among Andean countries, Ecuador has the second highest suicide rate, ahead of Colombia (6.1/100.000) and Peru (5.8/100.000) and behind Bolivia (18.7/100.000) [6, 22]. The reasons of this situation are not well understood; multifactorial risk factors might contribute to these differences among similar countries, and there may also be methodological differences in data collection and registering death causes.

It is not clear why some countries within the same region have significantly different suicide rates [6]. The World Health Organization reports that from the 800,000 people committing suicide each year, 50% are from China and India, and the highest rates reported were from Guyana, Lithuania, Hungary and South Korea [21, 33, 48]. Other countries including Saudi Arabia, Syria, Jordan, Nepal and Jamaica reported some of the lowest rates worldwide [49, 50].

The data from rural and urban areas showed different characteristics regarding the means of the suicidal act [51]. The incidence of suicide between rural and urban areas varies around the world due to their cultural, socioeconomic and geographical particularities [52]. In Ecuador, most of the reports come from the cities, more than doubling the cases from rural communities. Social stress factors such as high unemployment rates, social isolation, divorce, drug abuse, excessive working and negative chronobiological or physical working conditions have been identified as risk factors in the urban area [53–56]. Emotional conflicts occur in both rural and urban areas, but due to cultural and economic features, divorce as a reason for suicide is less frequent among the rural communities [57]. It has also been hypothesized that longer night shifts and busier lifestyles in urban areas can increase the risk of suicide [58]. There has been very little research on the effect of urbanization on the incidence of suicide. It has been identified that women perform more suicidal acts in urban areas than men, which may be due to job-related factors (quality, opportunity for promotions, etc.) and also vulnerability living in large cities [59].

It is not yet clear why men commit suicide more than women, despite knowledge that suicidal thoughts are more common in women than men, this proportion is observed across cultures. Certain factors have been identified that influence the suicide rate in men [60, 61]. For instance, the gender gap and the social pressure that is exerted on men because the image is generally linked to family support, which can cause economic failure to have a stronger negative impact on men. In Ecuadorian culture, men are commonly perceived or expected to be the primary head of household or even the family’s unique livelihood. They are expected to earn more than women counterparts so often times economic failure and feelings of inability to support one’s family can create an insurmountable pressure [62].

According to available literature, the means by which a man takes his own life appear to be more violent than those means utilized by women [17, 63]. In Ecuador, men use more violent methods than women, including death by firearms, hanging and sharp objects (Fig. 4). Interestingly, the landscape of means of suicide in Ecuador have changed in the last 15 years. Firearms used to account for 15% of the total causes of suicide in 2001, however in 2015, this number dropped to 5% (Fig. 5). This important reduction might be a consequence of better gun control and stronger national safety policy in Ecuador in the last 10 years [64]. For the first time ever in 2015 homicide rate dropped below the suicide rate, This trend might also be related with an important reduction in crime reported in Ecuador in the last years since suicide did not changed dramatically in the last 15 years. [65, 66].

An alarming discovery is that among adolescent women, suicide is the main cause of death, including all violent and non-violent causes. In the last years suicide has become a major public health problem among adolescents, specially in developed countries like the US [67]. Data from other countries shows that men adolescents are 2.6 times more likely to commit suicide than adolescent women [68]. In Ecuador, the proportion of suicide among adolescents account for more than 12% of the total causes of deaths, being this number higher in women (17%) than in men (10%). Similar reports have been published in other countries, showing that proportions are useful tools when estimating the impact of suicide among all other causes of the unnatural Death [69].

Suicide is usually preventable, especially in health systems where mental health services are readily available [70]. These preventable deaths need to be addressed to public health policy makers in order to plan and implement suicide prevention programs with a special focus on high-risk groups such as young adolescents women and single young men. Despite the fact that from 2001 to 2015 suicide rates in Ecuador were fairly stable, the health system and economic impacts are highly concerning due to premature mortality. Ecuador reported 4421 deaths registered as suicide, and the total years of healthy life lost exceeded 135,731 years. Using the human capital method, in 2001–2015 period, the total economic loss due to suicide was estimated to be $852.6 million international dollars (year 2010 base), almost 1% of the Ecuadorian gross domestic product (GDP) for 2015 [65].

### Limitations

The main limitation of our study is that data from suicide attempts was not available. In addition, the lack of data to assess confounding factors in order to better understand some of the observed associations and the possible causes of suicide is inexistent.

## Conclusions

This geodemographic study is the first of its kind in exploring the complete burden of suicide in Ecuador and its economic impact over the local health and productive system. In the last 15 years of available data, nearly 13,500 deaths were officially registered as suicide, putting the country above the regional average with a suicide rate of 7.1 per 100,000. Suicide largely is taking the life of younger people; especially adolescent women signifies that gender inequality may be a contributing factor. Some of the possible causes of higher suicide rate among younger people can be explained by chronic stressors such as family dysfunction, school failure, sexual abuse, alcohol, drug use and immigration patterns.

Despite the fact that some of the means of suicide has changed over time, the annual rate of suicide has not varied significantly in the last 15 years. In fact, while other means like firearms and pesticide consumption decreased in the last few years, hangings have increased over time.

Suicide seems to be more prevalent in high altitude provinces when compared to provinces at lower altitudes, however, this relationship does not demonstrate causality and further research is needed. Although this data is important, the analysis of risk factors and behavioral problems need to be addressed at a deeper level future studies.

The results of this study will provide an insight into the needs of authorities with regard to prevention programs and contributed to our understanding of suicide in Andean countries where information is scarce. Furthermore, it contributes to create the baseline data in order to publish guidelines for those involved in the medical practice of suicide specially those who are in charge of adolescents’ school-based education programs and gender inequality policies.

## Abbreviations

DALY: Disability-Adjusted Life Year
GBD: The Global Burden of Disease Group
ICD-10: International Classification of Diseases 10th revision
INEC: National Institute of Census and Statistics
IRB: Institutional Review Board
WHO: World Health Organization

## References

[1] Värnik P (2012). Suicide in the world. Int J Environ Res Public Health.

[2] Nordt C, Warnke I, Seifritz E, Kawohl W (2015). Modelling suicide and unemployment: a longitudinal analysis covering 63 countries, 2000–11. Lancet Psychiatry.

[3] Preti A (2007). Suicide among animals: a review of evidence. Psychol Rep.

[4] Jiménez-Ornelas RA, Cardiel-Téllez L (2013). El suicidio y su tendencia social en México: 1990-2011. Papeles Poblac.

[5] Brundtland GH (2001). Mental health: new understanding, new hope. JAMA.

[6] de la Salud. (OPS) OP. Mortalidad por Suicidio en las Américas: Informe Regional. Washington (DC); 2013.

[7] Global, regional, and national age–sex specific all-cause and cause-specific mortality for 240 causes of death, 1990–2013: a systematic analysis for the Global Burden of Disease Study 2013. The Lancet. 2015;385:117–71. 10.1016/S0140-6736(14)61682-2 PMC4340604 25530442

[8] Organization WH. Global health risks: mortality and burden of disease attributable to selected major risks [Internet]. World Health Organization; 2009 [cited 2014 Sep 14]. Available from: http://www.who.int/healthinfo/global_burden_disease/GlobalHealthRisks_report_full.pdf.

[9] Shojaei A, Moradi S, Alaeddini F, Khodadoost M, Barzegar A, Khademi A (2014). Association between suicide method, and gender, age, and education level in Iran over 2006–2010. Asia-Pac Psychiatry.

[10] Organization WH, others. Preventing suicide: A global imperative [Internet]. World Health Organization; 2014 [cited 2017 Mar 2]. Available from: http://apps.who.int/iris/handle/10665/131056.

[11] Bondy B, Buettner A, Zill P. Genetics of suicide. Mol Psychiatry. Nature Publishing Group; 2006 [cited 2017 Mar 2]. Available from: http://www.nature.com/mp/journal/v11/n4/full/4001803a.html.

[12] Hirschfeld RM, Russell JM (1997). Assessment and treatment of suicidal patients. N Engl J Med.

[13] Bolton JM, Pagura J, Enns MW, Grant B, Sareen J (2010). A population-based longitudinal study of risk factors for suicide attempts in major depressive disorder. J Psychiatr Res.

[14] Sinyor M, Schaffer A, Remington G (2015). Suicide in schizophrenia: an observational study of coroner records in Toronto. J Clin Psychiatry.

[15] Coope C, Donovan J, Wilson C, Barnes M, Metcalfe C, Hollingworth W (2015). Characteristics of people dying by suicide after job loss, financial difficulties and other economic stressors during a period of recession (2010–2011): a review of coroners′ records. J Affect Disord.

[16] Hawton K, Casañas I Comabella C, Haw C, Saunders K (2013). Risk factors for suicide in individuals with depression: a systematic review. J Affect Disord.

[17] Mueller AS, James W, Abrutyn S, Levin ML (2015). Suicide ideation and bullying among US adolescents: examining the intersections of sexual orientation, gender, and race/ethnicity. Am J Public Health.

[18] Lorenzo-Luaces L, Phillips JA (2014). Racial and ethnic differences in risk factors associated with suicidal behavior among young adults in the USA. Ethn Health.

[19] Brenner B, Cheng D, Clark S, Camargo CA (2011). Positive Association between Altitude and Suicide in 2584 U.S. Counties. High Alt Med Biol.

[20] Page A, Liu S, Gunnell D, Astell-Burt T, Feng X, Wang L (2017). Suicide by pesticide poisoning remains a priority for suicide prevention in China: analysis of national mortality trends 2006–2013. J Affect Disord.

[21] Barber CW, Miller MJ (2014). Reducing a suicidal person’s access to lethal means of suicide: a research agenda. Am J Prev Med.

[22] Teti GL, Rebok F, Rojas SM, Grendas L, Daray FM (2014). Systematic review of risk factors for suicide and suicide attempt among psychiatric patients in Latin America and Caribbean. Rev Panam Salud Publica.

[23] Mascayano F, Irrazabal M, Emilia WD, Shah B, Vaner SJ, Sapag JC (2016). Suicide in Latin America: a growing public health issue. Rev Fac Cienc Médicas.

[24] Sarmiento NMS, Proaño AMP, Stefos E (2016). Deaths by suicide in Ecuador: a quantitative data analysis. Rev Eur Stud.

[25] Gorenc K-D, Pacurucu S, Bruner C (2013). Estimación de la cifra verdadera del suicidio en Ecuador. Rev Psicol.

[26] González-Andrade F, López-Pulles R, Gascón S, García CJ (2011). Epidemiological issues regarding suicides in Ecuador: an 8-year report. J Public Health.

[27] Campo L (2015). Estudio paralelo del suicidio en el Ecuador como proceso ritual de significación. GRAFO Work Pap.

[28] INEC. Anuario de Estadisticas vitales: Nacimientos y Defunciones. 2001-2015. Available from: http://www.ecuadorencifras.gob.ec/nacimientos-defunciones/.

[29] Devleesschauwer B, Havelaar AH, De Noordhout CM, Haagsma JA, Praet N, Dorny P (2014). Calculating disability-adjusted life years to quantify burden of disease. Int J Public Health.

[30] Devleesschauwer B, McDonald S, Haagsma J, Praet N, Havelaar A, Speybroeck N. The DALY Calculator–a GUI for stochastic DALY calculation in R. 2014;

[31] Carlson RV, Boyd KM, Webb DJ (2004). The revision of the declaration of Helsinki: past, present and future. Br J Clin Pharmacol.

[32] ARCSA. Proceso de Autorización de Ensayos Clínicos a cargo de la Agencia Nacional de Regulación, Control y Vigilancia Sanitaria (ARCSA) [Internet]. 2017. Available from: http://www.controlsanitario.gob.ec/ensayos-clinicos/.

[33] WHO. Suicidio [Internet]. Centro de Prensa; 2017. Available from: http://www.who.int/mediacentre/factsheets/fs398/es/.

[34] Bertolote JM, Fleischmann A, Butchart A, Besbelli N (2006). Suicide, suicide attempts and pesticides: a major hidden public health problem. Bull World Health Organ.

[35] Kim N, Mickelson JB, Brenner BE, Haws CA, Yurgelun-Todd DA, Renshaw PF (2011). Altitude, gun ownership, rural areas, and suicide. Am J Psychiatry.

[36] Bertoli S, Marchetta F (2014). Migration, remittances and poverty in Ecuador. J Dev Stud.

[37] Virués-Ortega J, Garrido E, Javierre C, Kloezeman KC (2006). Human behaviour and development under high-altitude conditions. Dev Sci.

[38] Ortiz-Prado E, Dunn JF. High altitude exposure and ischemic stroke. Rev Fac Cien Med Quito 2011 [Internet]. 2011 [cited 2014 Mar 9];36:63–70. Available from: http://www.imbiomed.com.mx/1/1/articulos.php?method=showDetail&id_articulo=81935&id_seccion=3457&id_ejemplar=8088&id_revista=203.

[39] Kramer AF, Coyne JT, Strayer DL (1993). Cognitive function at high altitude. Hum Factors.

[40] Aquino Lemos V, Antunes HKM, Santos RVT, Lira FS, Tufik S, Mello MT (2012). High altitude exposure impairs sleep patterns, mood, and cognitive functions. Psychophysiology.

[41] INEC. VII Censo de Población y VI de Vivienda. 2010:2010.

[42] Krug EG, Mercy JA, Dahlberg LL, Zwi AB (2002). The world report on violence and health. Lancet.

[43] Leenaars AA (2006). Suicide among indigenous peoples: introduction and call to action. Arch Suicide Res.

[44] Boccagni P (2013). Migration and the family transformations it “leaves behind”: a critical view from Ecuador. Lat Am.

[45] Jokisch BD (2002). Migration and agricultural change: the case of smallholder agriculture in highland Ecuador. Hum Ecol.

[46] Ide N, Kõlves K, Cassaniti M, De Leo D (2012). Suicide of first-generation immigrants in Australia, 1974–2006. Soc Psychiatry Psychiatr Epidemiol.

[47] Milner A, McClure R, De Leo D (2012). Socio-economic determinants of suicide: an ecological analysis of 35 countries. Soc Psychiatry Psychiatr Epidemiol.

[48] Bunney WE, Kleinman AM, Pellmar TC, Goldsmith SK, others. Reducing suicide: A national imperative [Internet]. National Academies Press; 2002 [cited 2017 Mar 2]. Available from: https://www.ncbi.nlm.nih.gov/books/NBK220939/. 25057611

[49] FACPsychMed JBMD. Suicide prevention in Nepal: a comparison to Australia–a personal view. 2008 [cited 2017 Mar 9]; Available from: https://www.ncbi.nlm.nih.gov/pubmed/22477866. PMC2777576 22477866

[50] Madadin M, Mahmoud A, Alsowayigh K, Alfaraidy M (2013). Suicide deaths in Dammam, Kingdom of Saudi Arabia: retrospective study. Egypt J Forensic Sci.

[51] Searles VB, Valley MA, Hedegaard H, Betz ME. Suicides in urban and rural counties in the United States, 2006–2008. Crisis [Internet]. 2014 [cited 2017 Mar 9]; Available from: https://www.ncbi.nlm.nih.gov/pubmed/24067250. 10.1027/0227-5910/a000224 24067250

[52] Fontanella CA, Hiance-Steelesmith DL, Phillips GS, Bridge JA, Lester N, Sweeney HA (2015). Widening rural-urban disparities in youth suicides, United States, 1996-2010. JAMA Pediatr.

[53] Wang C-W, Chan CL, Yip PS (2014). Suicide rates in China from 2002 to 2011: an update. Soc Psychiatry Psychiatr Epidemiol.

[54] McCall TB (1988). The impact of long working hours on resident physicians. N Engl J Med.

[55] Amagasa T, Nakayama T, Takahashi Y (2005). Karojisatsu in Japan: characteristics of 22 cases of work-related suicide. J Occup Health.

[56] Baumert J, Schneider B, Lukaschek K, Emeny RT, Meisinger C, Erazo N (2014). Adverse conditions at the workplace are associated with increased suicide risk. J Psychiatr Res.

[57] Singh GK, Siahpush M (2002). Increasing rural–urban gradients in US suicide mortality, 1970–1997. Am J Public Health.

[58] Qin P, Agerbo E, Mortensen PB (2003). Suicide risk in relation to socioeconomic, demographic, psychiatric, and familial factors: a national register–based study of all suicides in Denmark, 1981–1997. Am J Psychiatry.

[59] Valencia JG, Montoya GJM, Jaramillo CAL, Tobón MCL, Guerra PM, Viana JCA (2011). Características de los suicidios de áreas rurales y urbanas de Antioquia. Colombia Rev Colomb Psiquiatr.

[60] Ladouceur R. Suicide among men [Internet]. The College of Family Physicians of Canada; 2011 [cited 2017 Mar 10]. Available from: http://www.cfp.ca/content/57/2/148.short.

[61] Rutz W, Rihmer Z. Suicide in men. Oxf Textb Suicidol Suicide Prev. 2009:249–55.

[62] Oquendo MA, Ellis SP, Greenwald S, Malone KM, Weissman MM, Mann JJ (2001). Ethnic and sex differences in suicide rates relative to major depression in the United States. Am J Psychiatry.

[63] Tsirigotis K, Gruszczynski W, Tsirigotis-Woloszczak M (2011). Gender differentiation in methods of suicide attempts. Med Sci Monit.

[64] García Gallegos B. La regulación de la seguridad privada en Ecuador: globalización, delincuencia y control civil de las Fuerzas del Estado. 2012 [cited 2017 Mar 10]; Available from: http://www.dspace.ups.edu.ec/handle/123456789/8530.

[65] The World Bank. Ecuador, data per country [Internet]. IBRD-IDA; 2014. Available from: http://data.worldbank.org/country/ecuador.

[66] DMQ. Seguridad ciudadana en Quito por parte del Observatorio de Seguridad Ciudadana [Internet]. Secretaría se Seguridad Metropolitana; 2016. Available from: http://vistazo.com/seccion/pais/la-tasa-de-suicidios-en-quito-supera-la-de-homicidios.

[67] Hawton K, Saunders KE, O’Connor RC (2012). Self-harm and suicide in adolescents. Lancet.

[68] McMahon EM, Keeley H, Cannon M, Arensman E, Perry IJ, Clarke M (2014). The iceberg of suicide and self-harm in Irish adolescents: a population-based study. Soc Psychiatry Psychiatr Epidemiol.

[69] Lukaschek K, Erazo N, Baumert J, Ladwig K-H (2012). Suicide mortality in comparison to traffic accidents and homicides as causes of unnatural death. An analysis of 14,441 cases in Germany in the year 2010. Int. J. Environ. Res. Public. Health.

[70] Crosby AE, Caine ED, Hindman J, Reed J, Iskander JK, Thorpe P, et al. Preventing suicide: a comprehensive public health approach. 2015 [cited 2017 Jul 19]; Available from: https://stacks.cdc.gov/view/cdc/34311.

